# Is lumbar facet joint tropism developmental or secondary to degeneration? An international, large-scale multicenter study by the AOSpine Asia Pacific Research Collaboration Consortium

**DOI:** 10.1186/s13013-016-0062-2

**Published:** 2016-02-09

**Authors:** Dino Samartzis, Jason Pui Yin Cheung, Shanmuganathan Rajasekaran, Yoshiharu Kawaguchi, Shankar Acharya, Mamoru Kawakami, Shigenobu Satoh, Wen-Jer Chen, Chun-Kun Park, Chong-Suh Lee, Thanit Foocharoen, Hideki Nagashima, Sunguk Kuh, Zhaomin Zheng, Richard Condor, Manabu Ito, Motoki Iwasaki, Je Hoon Jeong, Keith D. K. Luk, Bambang Prijambodo, Amol Rege, Tae-Ahn Jahng, Zhuojing Luo, Warat/Anant Tassanawipas, Narayana Acharya, Rohit Pokharel, Yong Shen, Takui Ito, Zhihai Zhang, Janardhana Aithala P., Gomatam Vijay Kumar, Rahyussalim Ahmad Jabir, Saumyajit Basu, Baojun Li, Vishal Moudgil, Ben Goss, Phoebe Sham, Richard Williams

**Affiliations:** Department of Orthopaedics & Traumatology, The University of Hong Kong, Pokfulam, Hong Kong, SAR China; Department of Orthopaedics, Ganga Hospital, Coimbatore, India; Department of Orthopaedic Surgery, University of Toyama, Toyama, Japan; Department of Orthopedics, Sir Gangaram Hospital, New Delhi, India; Spine Center, Wakayama Medical University, Kihoku Hospital, Ito-gun, Japan; Department of Spine Surgery, Eniwa Hospital, Hokkaido, Japan; Orthopaedic Department, Chang Gung Memorial Hospital, Taoyuan, Taiwan; Department of Neurosurgery, Seoul St. Mary’s Hospital, Catholic University of Korea, Seoul, South Korea; Department of Orthopedic Surgery, Samsung Medical Center, Sungkyunkwan University School of Medicine, Seoul, South Korea; Department of Orthopaedic Surgery, Khonkaen Regional Hospital, Khonkean, Thailand; Department of Orthopedic Surgery, Faculty of Medicine, Tottori University, Yonago, Japan; Department of Neurosurgery, Gangnam Severance Hospital, Seoul, South Korea; Department of Spine Surgery, The First Hospital Affiliated of Zhongshan University, Guangzhou, China; Department of Orthopedics, Cebu Orthopaedic Institute, Cebu, Philippines; Department of Advanced Medicine for Spine and Spinal Cord Disorders, Hokkaido University Graduate School of Medicine, Sapporo, Japan; Department of Orthopaedic Surgery, Osaka Rosai Hospital, Osaka, Japan; Department of Neurosurgery, College of Medicine, Soon Chun Hyang Unviersity Bucheon Hospital, Bucheon, South Korea; Department of Orthopaedic and Traumatology, Faculty of Medicine Airlargga University, Dr Soetomo Teaching Hospital, Surabaya, Indonesia; Department of Orthopaedics, Deenanath Mangeshkar Hospital, Jehangir Hospital, Pune, India; Department of Neurosurgery, Seoul National University Bundang Hospital, Seongnam, South Korea; Spine Service, Department of Orthopaedic Surgery, Xijing Hospital, the Fourth Military Medical University, Xi’an, China; Department of Orthopedics, Phramongkuthklao Army Hospital, Bangkok, Thailand; Dwaraka Institute of Spine Care, Bellary, India; Department of Orthopedics & Trauma Surgery, Spine Unit, Tribhuvan University, Teaching Hospital, Kathmandu, Nepal; Department of Spine Surgery, The Third Hospital of Hebei Medical University, Shijiazhuang, China; Department of Orthopaedic Surgery, Niigata City General Hospital, Niigata, Japan; Department of Orthopaedic Surgery, Beijing 361 Hospital (Aviation General Hospital), Beijing, China; Department of Orthopedics, Kasturba Medical College, Manipal University, Mangalore, India; Department of Neurosurgery, Fortis Hospital, Kolkata, India; Orthopaedic and Traumatology Department, University of Indonesia / RS Ciptomangunkusumo, Jakarta, Indonesia; Neurosciences Division, Park Clinic, Kolkata, India; Department of Orthopedic, Punjab Institute of Medical Sciences Jalandhar, Jalandhar, India; Department of Orthopaedics, University of Queensland, Brisbane, Australia

**Keywords:** Spondylolisthesis, Facet, Joints, Angulation, Orientation, Tropism, Developmental, AOSpine

## Abstract

**Background:**

Facet joint tropism is asymmetry in orientation of the bilateral facets. Some studies have shown that tropism may increase the risk of disc degeneration and herniations, as well as degenerative spondylolisthesis (DS). It remains controversial whether tropism is a pre-existing developmental phenomena or secondary to progressive remodeling of the joint structure due to degenerative changes. As such, the following study addressed the occurrence of tropism of the lower lumbar spine (i.e. L3–S1) in a degenerative spondylolisthesis patient model.

**Methods:**

An international, multi-center cross-sectional study that consisted of 349 patients with single level DS recruited from 33 spine institutes in the Asia Pacific region was performed. Axial MRI/CT from L3–S1 were utilized to assess left and right facet joint sagittal angulation in relation to the coronal plane. The angulation difference between the bilateral facets was obtained. Tropism was noted if there was 8° or greater angulation difference between the facet joints. Tropism was noted at levels of DS and compared to immediate adjacent and distal non-DS levels, if applicable, to the index level. Age, sex-type and body mass index (BMI) were also noted and assessed in relation to tropism.

**Results:**

Of the 349 subjects, there were 63.0 % females, the mean age was 61.8 years and the mean BMI was 25.6 kg/m^2^. Overall, 9.7, 76.5 and 13.8 % had L3–L4, L4–L5 and L5–S1 DS, respectively. Tropism was present in 47.1, 50.6 and 31.3 % of L3–L4, L4–L5 and L5–S1 of levels with DS, respectively. Tropism involved 33.3 to 50.0 % and 33.3 to 58.8 % of the immediate adjacent and most distal non-DS levels from the DS level, respectively. Patient demographics were not found to be significantly related to tropism at any level (*p* > 0.05).

**Conclusions:**

To the authors’ knowledge, this is one of the largest studies conducted, in particular in an Asian population, addressing facet joint tropism. Although levels with DS were noted to have tropism, immediate adjacent and distal levels with no DS also exhibited tropism, and were not related to age and other patient demographics. This study suggests that facet joint tropism or perhaps subsets of facet joint orientation may have a pre-disposed orientation that may be developmental in origin or a combination with secondary changes due to degenerative/slip effects. The presence of tropism should be noted in all imaging assessments, which may have implications in treatment decision-making, prognostication of disease progression, and predictive modeling. Having a deeper understanding of such concepts may further elaborate on the precision phenotyping of the facets and their role in more personalized spine care. Additional prospective and controlled studies are needed to further validate the findings.

## Background

**Fig. 1:**
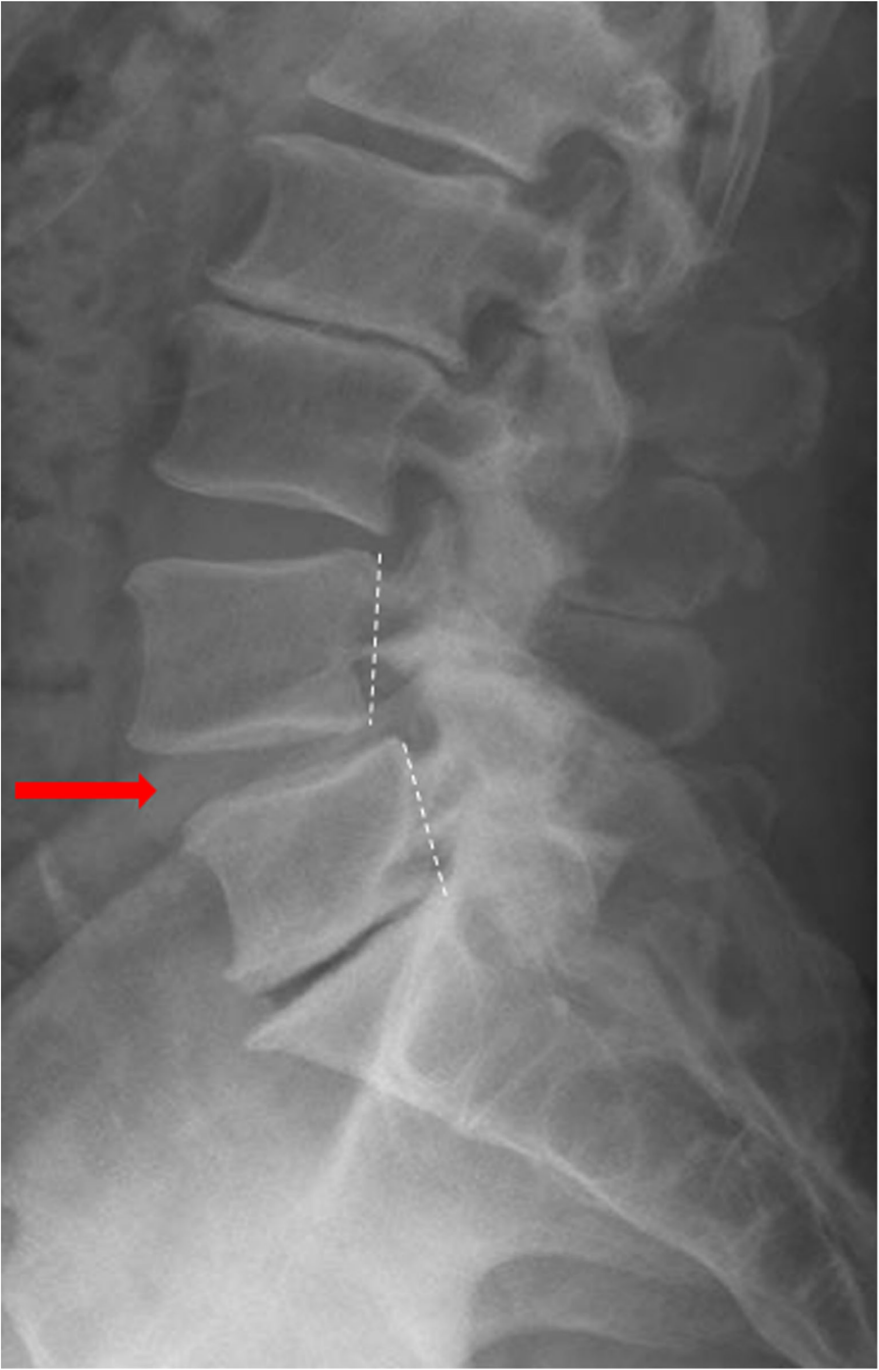
Lateral standing plain radiograph noting degenerative spondylolisthesis at L4–L5

**Fig. 2:**
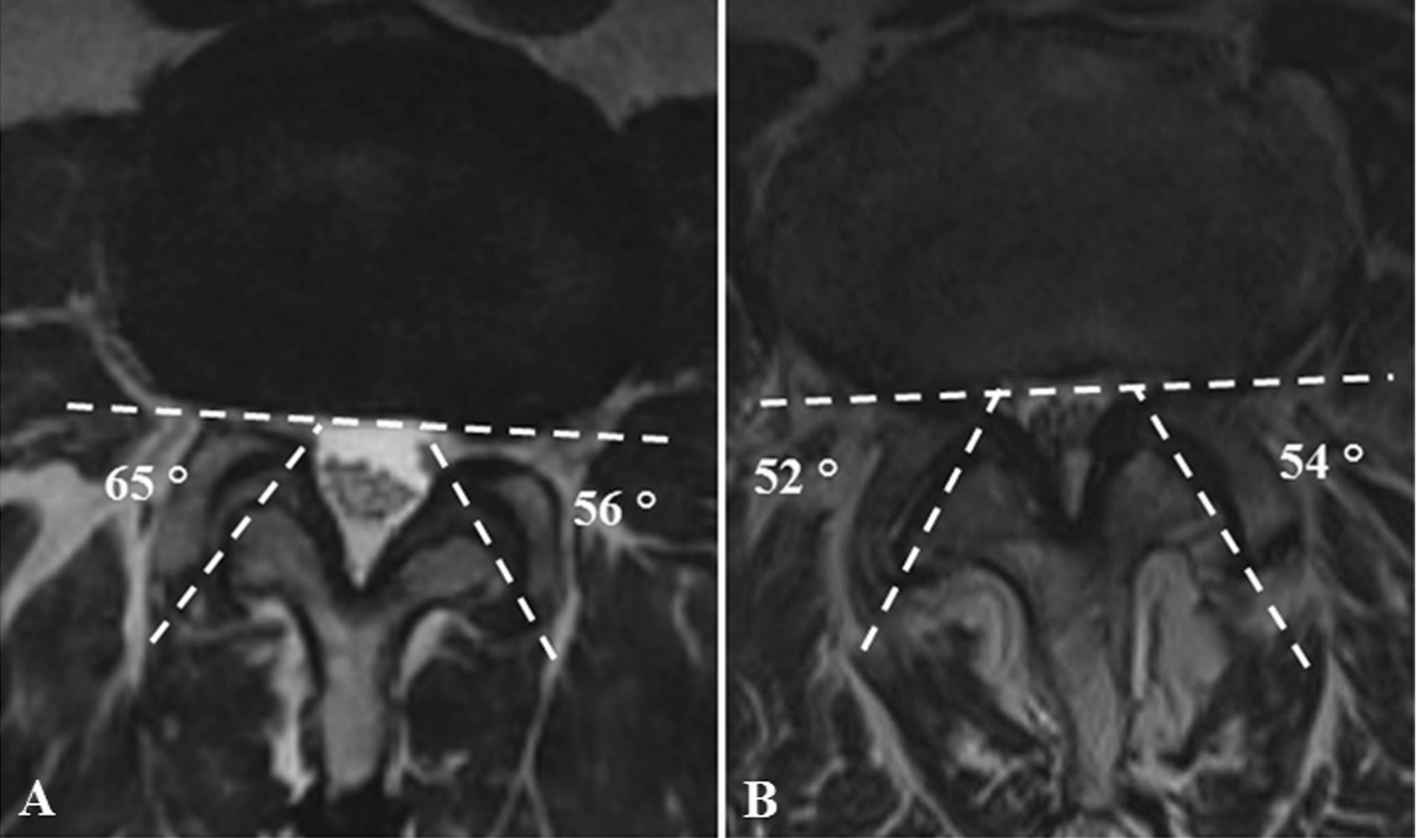
Axial MRI noting assessment of facet joint angulation. The images note patients with facet joint **a**) tropism and **b**) non-tropism. Asymmetry of the left and right facet joint angulations greater or equal to 8° angulation was defined as tropism

The lumbar facet joints are critical stabilizers of the motion segment preventing translation and excessive amounts of rotation and flexion [1, 2]. Approximately 33 % of the dynamic compressive load and 35 % of the static load are sustained by the facet joints [1, 2]. Degenerative spondylolisthesis (DS) is an outcome of facet joint dysfunction where one vertebral body is translated anteriorly in relation to the adjacent body, [3] mainly occurring at L4–L5 [4, 5] and in older age groups (Fig. 1) [6]. Such a condition may become symptomatic, often necessitating surgical intervention. Overall, increased sagittal alignment of the facet joints in relation to the coronal plane has been associated with the development of DS (Fig. 2). Even though increased facet joint angulation has been associated with DS, the role of facet joint angulation asymmetry, otherwise known as “tropism,” and the development of DS remains rather controversial [5].

Although facet joint orientation is critical in maintaining overall stability of the spine, the development of its angulation or tropism remains not well understood. It has “traditionally” been believed that disc degeneration of the spine, as in the setting of DS, may alter kinematics and load distributions, which may lead to secondary structural and morphological effects upon the facet joints and their orientation. In contrast to that belief, facet joint tropism may increase the motion and instability of a motion segment due to a destabilized posterior column [7–9]. With tropism, anterior shearing forces may not be well tolerated [7]. This may further increase the degenerative process in both the disc and the facet joints, thereby leading to DS [9–12].

While studies have indicated that facet joint tropism may manifest as a secondary cause following degeneration of the disc, some studies suggest that tropism may be a key risk factor for disc degeneration and herniation but the relationship may only be related to L4–L5, [13–17] which is also the most commonly affected level associated with DS. With regards to DS, tropism in these patients has been found to be greater than in normal subjects [18]. However, there are contradicting studies regarding this relationship [6, 19–21] and that tropism may not translate to facet joint degeneration [19]. There is still a lack of general understanding regarding how tropism develops, how it is defined and its clinical significance [22]. In addition, overall ethnic variations regarding facet joint orientation may exist [18, 21, 23–25].

Defining the role of facet joint tropism in the development of DS can improve our understanding of facet joint pathophysiology and the task of creating pathology-driven or more personalized management options. However, it remains controversial whether facet joint tropism is a pre-existing developmental phenomena or secondary to progressive remodeling of the joint structure due to degenerative changes. In theory, there could be individuals that may be pre-disposed to a specific facet joint angulation from inception that may further affect mechanics and either contribute to the onset or progression of disc degeneration. However, the concept of “developmental” origins to spine structures and their morphologies is an element that needs further exploration, but which already has some plausibility. For example, studies have shown that endplate abnormalities (e.g. Schmorl’s nodes) may increase the risk of disc degeneration and that some endplate defects may be painful [26]. Studies by Saluja et al. [27] and Dar et al. [28] have suggested that endplate abnormalities may be pre-existing. Luk and Samartzis [29] recently proposed the notion of disc “dysgeneration” whereby certain discs may have never fully developed or were healthy to begin to assume the status of a normal properly hydrated disc to degenerate in time, and as such should be regarded and classified differently. Such potential disconnect between dysgenerated and properly degenerated discs may account for the inability for many genetically-driven studies to identify reliable and replicated genes of disc degeneration because of misclassification of the degeneration phenotype [30]. With regards to facet joint angulation, Boden et al. [4] had suggested that in DS patients, largely based on a Caucasian population, that an increase in such angulation, not specifically tropism, may be attributed to anatomical variations and not a result of the DS process. Therefore, developmental origins of facet joint tropism may have some foundation that demand further exploration. As such, the following international multi-center study, initiated by the AOSpine Asia Pacific (AOSAP) Research Collaboration Consortium, addressed the occurrence of facet joint tropism of the lower lumbar spine (L3–S1) in a DS patient model within the Asia Pacific Region to determine if facet tropism occurred at levels with DS and at those adjacent to non-DS segments.

## Methods

### Study design and population

The study was an international, multi-center, cross-sectional radiographic study of DS patients in the Asia Pacific region with focus on facet joint tropism. Based on the AOSAP Research Collaboration Consortium, 33 international centers participated in this study [25, 31]. Ethics approval was obtained in all local institutional review boards before subject recruitment and patients provided consent to participate in the study. Study inclusion criteria included patients older than 18 years of age who were diagnosed with DS and were of Asian origin. Degenerative spondylolisthesis was diagnosed with a 3 mm or greater slip on lateral standing plain radiographs. Exclusion criteria included patients with previous or current spinal surgery, congenital anomalies, transitional vertebrae, previous infection, trauma, tumors, isthmic spondylolisthesis, and unsatisfactory imaging. There were 371 patients with known ethnic origin. Of these individuals, 349 patients were included in this study with complete data parameters and who had single level DS at any segment from L3–S1.

### Imaging assessment

Standing lateral plain radiographs and sagittal/axial T2-weighted lumbar magnetic resonance images (MRI) of the lumbar spine were obtained. Axial MRIs were selected based on the level that closely bisected the facet joints at each segmental level. Imaging cut sequences were at least 3 mm. Magnetic resonance imaging slices were preferred if they included the posterior/superior corner of the caudal vertebral body. This was the slice which most closely bisected the facet joint and was utilized for measuring the facet joint geometry. If this exact slice was not available from the scans performed, the most closely situated slice was used. If the selected slice was more than 2 mm cranial or caudal to the ideal slice cut, a new scan was ordered.

The axial MRIs from L3-S1 were used to measure the left and right facet joint angulation in relation to the coronal plane. The angulation degree was obtained based on the intersecting line of the posterior border of the vertebral body in the coronal plane to that of the line bisecting the inferior and superior tips of the facet joint process (Fig. 2). The difference in angulation between the left and right facet joints was obtained to calculate tropism. Based on the description by Samartzis et al., [31] facet joint tropism was defined as angulation difference of ≥8° in sagittal orientation between the left and right facet joint angles (Fig. 2). An independent observer who was not participating in the clinical management of these patients assessed all the imaging. The imaging protocol has been previously reported in greater detail [25, 31]. In addition, patient demographic information was obtained of each patient, which included age (years), sex-type, weight (kg), height (meters), BMI (kg/m^2^) and ethnicity. Although ethnicity was documented, it did not form the basis of this study for it was addressed as an independent variable in previous work [25].

### Statistical analyses

All data was anonymized and coded. SPSS version 23 statistical software (Chicago, IL) was utilized to perform the statistical analyses. Univariate analysis was conducted of the data of interest. Descriptive and frequency analyses were performed, in particular to assess the prevalence of facet joint tropism at the DS level and in relation to its adjacent level(s). The threshold of statistical significance was noted with *p*-values ≤0.05.

## Results

Of the 349 subjects with single level DS, 63 % were females and 37 % were males. The mean age was 61.8 years (range: 24.0–90.0; ±SD: 12.4 years) and the mean BMI was 25.6 kg/m^2^ (range: 15.4–43.9; ±SD: 4.2 kg/m^2^). Degenerative spondylolisthesis involved 9.7 % of L3–L4, 76.5 % of L4–L5 and 13.8 % of L5–S1 levels. Overall, 78 patients had no (22.3 %) levels with facet joint tropism; whereas, 121 (34.7 %), 100 (28.7 %) and 50 (14.3 %) patients had 1, 2 or 3 levels of tropism, respectively.

**Fig. 3:**
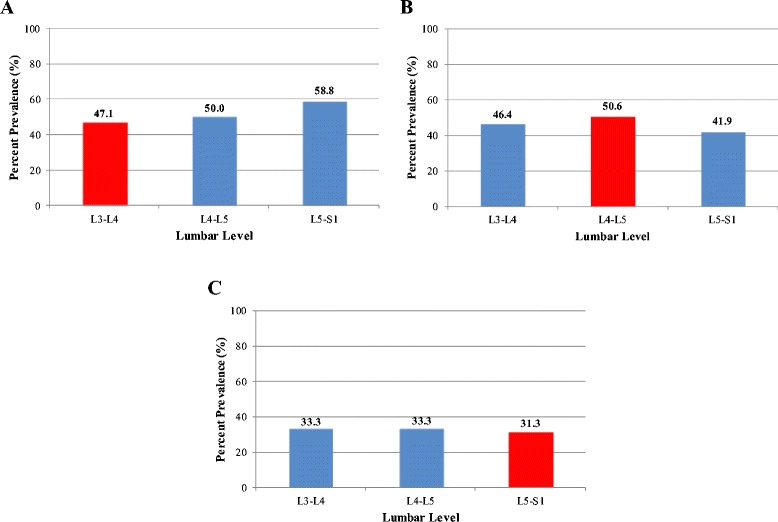
Percent prevalence of facet joint tropism of the lumbar spine in patients with degenerative spondylolisthesis of **a**) L3–L4, **b**) L4–L5, and **c**) L5–S1. * Note that the red bar indicates the level of spondylolisthesis

With regards to DS at L3–L4 (Fig. 3a), there were 34 patients of which 58.8 % were females. The mean age was 60.8 years (range: 38.0–82.0; ±SD: 11.0 years) and the mean BMI was 24.1 kg/m^2^ (range: 15.6–34.8; ±SD: 4.2 years). Tropism involved 47.1 % of all L3–L4 DS levels. Tropism was also noted in 50.0 and 58.8 % at the immediate (L4–L5) and distal (L5-S1) non-DS levels, respectively. Overall, in patients with L3-L4 DS, 11.8, 32.4, 44.1 and 11.8 % had 0, 1, 2 or 3 levels with tropism, respectively.

With respect to DS at L4–L5 (Fig. 3b), there were 267 patients of which 64.4 % were females. The mean age was 63.2 years (range: 28.0–90.0; ±SD: 11.6 years) and the mean BMI was 25.8 kg/m^2^ (range: 17.3–43.9; ±SD: 4.2 kg/m^2^). Tropism was present in 50.6 % of all L4–L5 DS levels. Tropism also involved 46.4 and 41.9 % of the immediate adjacent non-DS levels of L3–L4 and L5–S1, respectively. As a whole, in patients with L4–L5 DS, 22.5, 33.0, 27.7 and 16.9 % had 0, 1, 2 or 3 levels with tropism, respectively.

In individuals with L5-S1 DS (Fig. 3c), there were 48 patients (58.3 % females). The mean age was 54.3 years (range: 24.0–79.0 years; ±SD: 14.9 years) and the mean BMI was 25.5 kg/m^2^ (range: 15.4–36.5; ±SD: 4.1 kg/m^2^). Tropism was noted in 31.3 % of all L5–S1 DS levels. Tropism was also noted in 33.3 and 33.3 % at the immediate (L4–L5) and distal (L3–L4) non-DS levels, respectively. Overall, in patients with L5-S1 DS, 29.2, 45.8, 22.9 and 2.1 % had 0, 1, 2 or 3 levels with tropism, respectively.

Facet joint tropism was most prevalent in DS levels with L4–L5 involvement. Patients with L4–L5 DS had more levels with tropism involvement than L3–L4 or L5–S1 with DS. Age, sex-type and BMI were factors that were not significantly related to any level (*p* > 0.05).

## Discussion

To our knowledge, our study was one of the largest international studies, particularly focusing on an Asian population, addressing the role of facet joint tropism in relation to lumbar levels with DS and its occurrence at adjacent segments. Findings from the study indicated that tropism was present more often at the level of a L4–L5 DS than at its non-DS levels. Similar tropism rates were noted at adjacent levels in relation to a L5–S1 DS and at higher rates at adjacent levels in relation to a L3–L4 DS. However, tropism was also noted in the immediate and distal adjacent non-DS levels in relation to the DS segment, ranging in prevalence from 33 to 60 %. More specifically, in the setting of DS levels with L3–L4 or L5–S1, the immediate adjacent and more distal levels had similar tropism rates between each other. Additional analysis also showed no relationship between tropism with patient demographics, such as age, sex-type and BMI.

As described by Kirkaldy-Willis et al., [32] the spine’s degenerative cascade begins with intervertebral disc degeneration, which is more prevalent with increasing age. These degenerative changes further alter the biomechanics of the motion segment. As a result, it has been propagated that the facet joints are overloaded and become more susceptible to anterior shearing forces leading to facet joint remodeling and the development of DS. However, the role of facet joint tropism upon the development of DS remains controversial. According to a systematic review by Devine et al., [22] the authors reported no significant relationship between tropism and DS. This finding can be contributed to multiple factors related to tropism, such as inconsistencies with the definition of the phenotype, variations in age, ethnicity and biomechanical factors [19, 31]. However, in a recent study by Samartzis et al. [31] assessing the role of facet joint tropism in a large-scale Asian population with or without L4–L5 DS, facet joint tropism was significantly associated with DS.

Overall, uncertainty still exists surrounding the interactions between facet joint tropism, disc degeneration and DS. The natural course of the facet joints are largely unknown. From one perspective, tropism can be a remodeling manifestation secondary to disc degeneration and rotational instability of the spine [9–12]. Alternatively, some studies report no relationship between disc degeneration and tropism, [6, 19–21] which would suggest its presence to have a more developmental origin. As previously noted in this article, evidence exists to a potential “developmental” component of disc degeneration and endplate abnormalities [27–29, 33]. As such, a developmental variety of facet joint angulation, manifesting in subsets of tropism, may also exist, which may increase the risk of clinically relevant conditions (e.g. DS). The current study has noted that such tropism is present in lumbar levels with and without DS, which is contrary to traditional thought that such facet orientation is secondary to remolding changes as a result of the DS. Therefore, such work lends further credence as to an alternative chain of events to the long-held belief of the degenerative cascade of the spine in that perhaps the facet joints may directly or indirectly play a role in degenerative disc changes that may further alter kinematics and loading, thereby further affecting the posterior column and increasing susceptibility to anterior sheer forces of the motion segment that may eventually lead to a DS. However, the presence of tropism at other levels without DS doesn’t exclude that it is secondary to DS. In fact, in some individuals, this could be a combination of developmental and secondary changes.

As with any clinical and multi-center study, inherent limitations exist. A matched-control group consisting of individuals with no DS at any level was not available for direct comparisons. However, within-subject lumbar levels of non-DS segments were used as comparative controls. As such, we accounted for facet joint tropism at the adjacent and most distal levels, when applicable, in relation to the DS level to minimize any perceived immediate adjacent compensatory hypermobility effects by the index DS segment [34]. Such assessment yielded consistent findings in comparison to the immediate and most distal adjacent segments in relation to the DS level. In addition, the generalizability of the study still needs to be assessed since our study population was composed of Asian subjects. However, due to the heterogeneic nature and potential confounds accompanying multi-ethnic studies, we found that focusing on a purely Asian population may minimize potential confounding factors regarding ethnicity. Furthermore, our previous work also noted that facet joint angulations did not significantly differ between various Asian ethnicities [25]. In addition, this study was cross-sectional in nature, whereby future prospective, longitudinal and multi-modal imaging studies are needed to assess the precise cause and effect estimate of tropism upon other spinal phenotypes, such as disc degeneration, endplate changes, alignment alterations, and the development of DS. However, since tropism was noted in levels without DS, in particular in regions where disc degeneration effects are often not as pronounced (e.g. L3–L4), it can be assumed that for certain individuals tropism may be a pre-existing factor, independent of DS. However, as previously mentioned, some individuals may have combined developmental and secondary changes effecting the facet joints, which demands further exploration. Such concepts may need to also re-visit and re-emphasize degeneration-related tropism to that of slip-related tropism.

## Conclusions

To the authors’ knowledge, this is the largest study to date that addresses the role of facet joint tropism and its association with lumbar levels with DS in comparison to their adjacent non-DS levels in an Asian population. Our study noted that L4–L5 levels with DS had a higher prevalence of tropism than other DS levels; however, tropism was noted in non-DS levels adjacent and more distally to the DS segment and was independent of age. Such findings suggest that facet joint tropism may, in some individuals, be a pre-existing phenotype, which would deem further investigation. Furthermore, there could be individuals that may have a “combination” of developmental and secondary changes from degeneration or the vertebral slip that may affect the facet joints. Tropism may play an instrumental role in treatment decision-making, prognostication of disease progression and predictive modeling; as such, the authors suggest that the presence of tropism on image assessment should be noted.

In an age where genomics and other “omics” approaches have gained widespread applicability towards better understanding disease, having an improved understanding of spinal phenotypes, such as facet joint orientation, may further shed light as to the pathogenesis of a spinal condition and help in developing early-recognition, preventative measures and tailored management options. Although the current study is cross-sectional in nature, future prospective studies are needed to more robustly assess if facet joint orientation, specifically tropism, is developmental in origin, secondary to the remodeling process of degeneration/slip, or a combination of both. Nonetheless, this study further raises awareness of the issue of a potential developmental component to facet joint orientation that may have clinical implications, stressing the need for future studies.
